# Acute khat use reduces response conflict in habitual users

**DOI:** 10.3389/fnhum.2013.00285

**Published:** 2013-06-19

**Authors:** Lorenza S. Colzato, Roberta Sellaro, Manuel J. Ruiz, Katarzyna Sikora, Bernhard Hommel

**Affiliations:** ^1^Institute for Psychological Research, Leiden Institute for Brain and Cognition, Leiden UniversityLeiden, Netherlands; ^2^Department of Psychology, Granada UniversityGranada, Spain

**Keywords:** khat, response conflict, Simon task, dopamine, interference control

## Abstract

Khat consumption has become a worldwide phenomenon broadening from Eastern Africa and the south west of the Arabian Peninsula to ethnic communities in the rest of the world. So far, the cognitive effects of khat use are poorly understood and no studies have looked into the relation between acute khat use and cognitive control functions, the way we control our thoughts and goal directed behavior. We studied how acute khat use affects the emergence and the resolution of response conflict, a central cognitive control function. Khat users (*n* = 11) and khat-free controls (*n* = 18) were matched in terms of education, sex, alcohol, and cannabis consumption. Groups were tested on response conflict, as measured by the Simon task. In one single session, participants worked through two task blocks: the khat group chewed exclusively khat whereas the khat-free group chewed solely a gum. Results showed that in the second block, which reflects the acute impact of khat, the khat group was better than controls in resolving stimulus-induced response conflict as indexed by a smaller Simon effect. These results suggest that the acute intake of khat may improve participants' ability of handling response conflict.

## Introduction

Chewing leaves from the khat plant (*Catha Edulis*) in Eastern Africa and in the south west of the Arabian Peninsula is a tradition dating back hundreds of years. During the last 10 years, mainly because of the Somali diaspora, chewing khat has turned into a worldwide phenomenon broadening to ethnic communities in the rest of the world, such as in North America, Great Britain, and the Netherlands (UNODC, 2010). Chewing khat is a more frequent habit for men than it is for women, often with the aim to enhance social interaction. It is used in informal meetings (khat sessions), in which the participants maintain social contact and experience alertness, it reduces hunger and enhances self-esteem (Brenneisen et al., 1990; Kalix, 1996). During those sessions, the leaves and the tender younger stalks of the plant are chewed slowly over several hours.

So far, studies have only systematically looked into the detrimental effect of chronic use of khat on cognition. First, Colzato et al. (2011a) reported that chronic khat users (*n* = 20), in a stop-signal paradigm, exhibit impairments in the inhibition of behavioral responses compared to khat-free controls (*n* = 20). Participants were asked to press a left or right button as soon as a green left- or right-pointing arrow appeared (go trials). However, if the color of the arrow suddenly changed to red, the participants were supposed to refrain from responding (stop-trials). On go trials, chronic khat users performed just as well as non-users in terms of both accuracy and response speed. However, chronic khat users were significantly slower than non-users in inhibiting on time on stop-trials.

Second, Colzato et al. (2011b), testing the same pool of participants as in the previous study (Colzato et al., 2011a), showed that chronic khat users (*n* = 20) performed significantly worse than controls (*n* = 20) on the global-local task-switching paradigm (an index of cognitive flexibility) and on the N-back task (an index of working memory updating). In the task-switching paradigm participants were confronted with hierarchical geometrical figures (large squares or rectangles made of small squares or rectangles) and were supposed to switch every four trials between responding to the global and responding to the local target dimension. In the global task, participants were to react to the global shape of the stimulus (i.e., the large square or rectangle, irrespective of the shape of its local components), whereas in the local task they had to respond to the component shapes that constituted the large shape. Chronic khat users showed more pronounced switching costs [i.e., a greater difference in reaction times (RTs) between alternation trials and repetition trials] than khat-free controls, an indication of decreased cognitive flexibility. In the N-back task, participants were confronted with a stream of letters and had to indicate whether the present letter matched the one that was presented directly before (1-back) or in the second-to-last trial (2-back). Chronic khat users committed significantly more errors in both the 1-back and 2-back conditions, an indication of more deficient WM updating.

Third, Colzato et al. (2012), testing a new pool of participants, showed that chronic khat users (*n* = 16) displayed less efficient resolution of response conflicts as indexed by the Simon task than khat-free controls (*n* = 16). In this task, participants respond to a non-spatial feature of commonly visual stimuli (e.g., color) by pressing left and right response buttons. Importantly, the location of the stimulus varies randomly, so that it can spatially correspond, or not correspond with the correct response. As one might expect, performance is better with stimulus-response correspondence than with non-correspondence—the Simon effect (Simon and Small, 1969). The effect reflects the difficulty of selecting a response in the face of competing response tendencies and can thus be taken as a rather pure measure of (the efficiency of resolving) response conflict (Kornblum et al., 1990; Hommel, 2011). Compared to khat-free controls, chronic khat users were more strongly affected by stimulus-induced response conflict. Moreover, the hours chewing khat positively correlated with the size of Simon effect, indicating that longer chewing was associated with increased response conflict.

The active ingredients of kath are cathine and cathinone, the second of which is the main contributor of the psychostimulant effect of khat. These alkaloids are similar in structure and pharmacological activity to amphetamines (Wagner et al., 1982): both stimulate the central nervous system (CNS) and suppress appetite. For this reason khat has gained the reputation as a “natural amphetamine.” Compared to amphetamine, the onset of action of cathinone is faster (15 min.) (Cox and Rampes, 2003) while the half life is shorter (90 min.) (Patel, 2000). Cathinone increases levels of dopamine (DA) and norepinerphrine (NE) in the brain by acting on the catecholaminergic synapses, delaying the reuptake and/or enhancing the release of those neurotransmitters (Wagner et al., 1982; Patel, 2000). In particular, DA has been considered the key neurotransmitter in driving cognitive control, the way humans control their thoughts and goal directed behavior (Cools, 2006).

Remarkably, the use of synthetic cathinones, such as mephedrone and butylone, has increased among individuals who go clubbing, at least in UK (Wood et al., 2011). Similarly to khat, at high doses, mephedrone can induce unpleasant side effects, such as hallucinations, sweating, headache, palpitations, nausea, and vomiting.

Keeping in mind the similarity between cathinone and amphetamine, the results of our three pervious studies (Colzato et al., 2011a,b, 2012) are in line with studies showing impairments in WM (Daumann et al., 2004), response inhibition and cognitive flexibility (van der Plas et al., 2009) and in response conflict (Rubia et al., 2011) with chronic amphetamine and methamphetamine use. The current study focused, for the first time, on the acute effect of khat on this latter key cognitive control function: the ability to deal with, and resolve response conflict, that is, the ability to select a correct response in the face of other, competing response tendencies.

Interestingly, performance on the Simon task seems to be modulated by DA (Onur et al., 2011), one of the two key neurotransmitters augmented during the acute use of khat. Holroyd and Coles (2002) argued that, when a behavioral error is executed, a phasic dip in activity of midbrain DA neurons “passes on” a prediction-error signal as a warning that the actual behavioral output is worse than expected. This warning signal is transferred to the anterior cingulate cortex (ACC) where it gives rise to adaptations in response selection by modifying motor programs in order to optimize performance for the task on hand.

In the present study we tested whether acute khat use, in contrast to chronic use, facilitates the resolution of response conflict indexed by the Simon task (Simon and Small, 1969). Given the above-mentioned relation between DA and response conflict and ACC on the one hand, and between DA and acute khat use on the other, we expected reduced response conflict, as indicated by a smaller Simon effect, in khat users as compared to khat-free controls. To ascertain that this effect is produced by khat and not by the simple activity of chewing: we had the khat group chewing exclusively khat whereas the khat-free group chewing solely a gum while performing the Simon task. If the activity of chewing is responsible for the reduced Simon effect, the effect would be equally reduced in both groups. Participants in both groups worked through two task blocks. In the khat group the first block, which was considered the drug-free baseline performance, took 15 min. to perform, that is, before the onset of action of khat. Accordingly, in the khat group, the second task block was considered to reflect the acute impact of khat, which will be indexed by reduced response conflict. In the khat-free group, which chewed only a gum, we did not expect, instead, any difference of response conflict between the task blocks.

## Materials and methods

### Participants

Thirty young healthy adults (27 men), who never participated in previous behavioral pharmacology studies, were compensated for their participation. They constituted the two groups of 12 khat users and 18 khat-free controls. The sample was drawn from 40 adults in the Leiden and The Hague metropolitan area, who volunteered to participate in studies of behavioral pharmacology. Participants were recruited via ads posted on community bulletin boards and by word of mouth. Participants were selected via an interview using the Mini International Neuropsychiatric Interview (MINI; Sheehan et al., 1998). The MINI is a well-established brief diagnostic tool in clinical, stress and psychopharmacology research (Sheehan et al., 1998; Elzinga et al., 2008; Colzato et al., 2009) that screens for several psychiatric disorders including, post-traumatic stress disorder, schizophrenia, depression, mania, ADHD, and obsessive-compulsive disorder. Based on the interview, we excluded 10 of the 40 potential participants because of current medication use. All khat users met more than four out of the seven criteria that according to the American psychiatric Association DSM-IV and the World Health Organization (ICD-10) define addiction: tolerance, withdrawal, difficulty controlling the use, negative consequences for job, family and health, significant time or emotional energy spent in searching/consuming the drug, put off or neglected activities because of the use, and desire to cut down the use.

We made sure that the users met the following criteria: (1) khat consumption by chewing route for a minimum of 1 year; (2) no clinically significant medical disease; and (3) no use of medication.

None of the khat-free controls reported any history of past or current khat use.

Following Colzato et al. (2011a,b, 2012), participants were asked to refrain from taking any psychoactive drugs for at least 24 h before the test, not to consume alcohol on the night before the experimental session, and to have a normal night rest. Participant's compliance with the instruction was encouraged by taking a (not further analyzed) saliva sample test at the beginning of the session (cf. Colzato et al., 2004, 2009).

The two groups were matched for sex, education (every subject being an undergraduate student or possessing a bachelor or a master degree) and alcohol and cannabis consumption. Even though khat was the preferred drug for users, some of them drank alcohol: either on weekly base often in combination with cannabis or on a monthly base. Khat users and non-users reported to have never used LSD, MDMA, cocaine, amphetamine, barbiturates, ketamine, GHB or speed. Demographic and drug use information are provided in Table 1. Written informed consent was obtained from all participants after the nature of the study was explained to them. The protocol and the remuneration arrangements of 15 Euro were approved by the institutional review board (Leiden University, Institute for Psychological Research).

**Table 1:** **Demographic characteristics and self-reported use of khat and other psychoactive drugs**.

**Sample**	**Khat users**	**Khat-free controls**
*N* (M:F)^n.s.^	11 (11:0)	18 (15:3)
Age (years)^**^	31.5 (5.4)	20.8 (3.0)
Khat exposure (years)^**^	8.8 (5.2)	0
Khat times in a week^**^	3.3 (1.7)	0
Bundles used (khat shrubs)^**^	3.6 (1.8)	0
Bundles used in one session	3.6 (1.8)	0
Hours chewing khat^**^	6.2 (2.1)	0
Last time khat use (in hours)	30 (6.0)	0
Weekly cigarettes	10 (9.8)	11 (24.1)
Monthly exposure (joints)^n.s.^	1.2 (3.1)	0.9 (1.9)
Monthly drinks (units)^n.s.^	0.6 (1.5)	1.4 (1.1)
Lifetime cocaine (grams)^n.s.^	0	0
Lifetime amphetamines (grams)^n.s.^	0	0
Lifetime ketamine (grams)^n.s.^	0	0
Lifetime speed (grams)^n.s.^	0	0

Standard deviations are presented within parentheses.

Bundles used, number of khat bundles consumed in a typical day/session; Hours chewing khat, amount of time the users spend chewing khat in a typical day/session; Monthly drinks, monthly number of standard alcoholic drinks.

n.s. Non-significant difference.

Significant group difference,

** p < 0.01.

### Cognitive task

The Simon task has been previously employed to systematically investigate the neuroplastic effects of chronic khat use (Colzato et al., 2012). The experiment was adapted from Hommel (1993) and consisted of a 35 min. session in which participants made speeded discriminative button-press responses to the pitch of a tone. Participants responded left to a low tone (200 Hz) and right to a high tone (800 Hz). Tones were equiprobably presented to the right or to the left ear by means of earphones until the response was given or 2000 ms had passed. Participants were to ignore the location of the ear in which the tone was presented and to base their response exclusively on its pitch. Responses were to be given as fast as possible while keeping error rates below 15% on average; feedback was provided at the end of a trial block. The task consisted of 1 practice block of 20 trials and 2 experimental blocks (15 min. each) of 240 trials (with all conditions being equiprobable).

### Procedure and design

All participants were tested individually. Participants provided a saliva sample, and subsequently performed the behavioral task measuring response conflict. Participants were tested in one single session (35 min.): after the practice block (5 min.) in which no chewing was required, participants in the khat group chewed exclusively khat when performing the two task blocks of the duration of 15 min. each. All khat users were provided by the experimenter with the same amount and type of fresh khat leaves (50–75 g). Participants were encouraged to chew khat leaves during 8 brief breaks scheduled every 60 trials, till the amount was finished. Participants in the khat-free group followed the same procedure; however, after the practice block instead of chewing khat they solely chewed gum during the execution of the task blocks. Participants were required to chew 1–2 (sportlife) gums provided by the experimenter and they were let free to choose among three different flavors: mint, tropical twist, or strawberry. For both groups no break was allowed between task blocks.

The participants were tested by a lab-assistant blind to the hypothesis being tested. The experiment was controlled by a PC attached to a 17″ monitor with a refresh rate of 120 Hz.

### Statistical analysis

Independent *t*-tests were performed to test age, alcohol, and cannabis consumption differences between the groups.

In the Simon task, after eliminating the practice block, mean correct RTs (excluding anticipations, that is, RTs faster than 200 ms) and error percentages (PEs) were analyzed by means of repeated-measures ANOVAs using spatial Stimulus-Response Correspondence (vs. non-correspondence) and Block (1 vs. 2) as within- and Group as between-participants factor. Given that the onset of action of khat is 15 min. after starting chewing, which corresponds to the duration of the first task block, we expected khat, in the khat group, to reduce the Simon effect only in the second task block. This effect would be statistically indicated by a significant three-way interaction involving spatial Stimulus-Response Correspondence, Block, and Group.

Moreover, partial correlations controlling for age were computed between the degree of exposure to khat and the difference in Simon effect from block 1 to block 2, in order to test whether in block 2 the magnitude of cognitive enhancement is proportional to the amount of khat consumed.

Effect sizes were assessed by calculating partial Eta squared (η^2^_*p*_) for repeated-measures ANOVAs. The data of one male khat-user were excluded because of his excessive error rate (PE > 40%). A significance level of *p* < 0.05 was adopted for all tests.

## Results

### Participants

No significant group differences were obtained for alcohol consumption, *t*_(27)_ = 1.53, *p* = 0.14, or cannabis consumption, *t*_(27)_ = 0.26, *p* = 0.80, but for age, *t*_(27)_ = 6.92, *p* < 0.001: individuals in the khat group were significantly older than in the khat-free group. Table 1 shows demographic characteristics and drug-use profiles for the two groups.

### Simon task

The RT analysis showed a Group effect, *F*_(1, 27)_ = 5.95, *p* = 0.02, Mean Square Error (MSE) = 42964.62, η^2^_*p*_ = 0.18: khat users were in general slower than khat-free controls (562 vs. 465 ms). Khat users also tended to be less accurate than khat-free controls (5.4% vs. 3.5%), *F*_(1, 27)_ = 3.68, *p* = 0.07, MSE = 26.92, η^2^_*p*_ = 0.12. The RT and PE analyses showed a main effect of Correspondence, *F*_(1, 27)_ = 125.13, *p* < 0.001, MSE = 762.53, η^2^_*p*_ = 0.82 (RTs) and *F*_(1, 27)_ = 61.54, *p* < 0.001, MSE = 9.43, η^2^_*p*_ = 0.70 (PEs), as indication that responses were faster and more accurate with stimulus-response correspondence (484 ms and 2.2%) than with non-correspondence (543 ms and 6.8%, respectively). The main effect of Correspondence was modified by Block and Group in RTs but not in errors, *F*_(1, 27)_ = 5.42, *p* = 0.03, MSE = 367.08, η^2^_*p*_ = 0.17 (RTs) and *F* < 1 (PEs). Table 2 shows the results of ANOVAs performed on RTs and PEs. To disentangle the interaction, we ran separate ANOVAs for the two blocks (with Correspondence and Group as independent variables) and for the two groups (with Correspondence and Block as independent variables).

**Table 2:** **Results of analysis of variance on mean reaction time of correct responses (RT) and percentage of errors (PE)**.

		**RT**		**PE**	
**Effect**	***df***	**MSE**	***F***	**MSE**	***F***
Group (G)	27	42964.62	5.95, *p* = 0.02^*^	26.92	3.68, *p* = 0.07
Block (B)	27	4054.73	13.53, *p* = 0.001^**^	6.41	2.23, *p* = 0.15
Correspondence (C)	27	762.53	125.13, *p* = 0.0001^***^	9.43	61.54, *p* = 0.0001^***^
G × B	27	4054.73	0.51, *p* = 0.48	6.41	0.60, *p* = 0.44
G × C	27	762.53	0.11, *p* = 0.74	9.43	0.01, *p* = 0.93
B × C	27	367.08	7.91, *p* = 0.009^**^	5.21	5.08, *p* = 0.05^*^
G × B × C	27	367.08	5.42, *p* = 0.03^*^	5.21	0.37, *p* = 0.58

* p < 0.05;

** p < 0.01;

*** p < 0.001.

In block 1, the Correspondence and Group interaction was not significant, *F* < 1. Thus, even if there was a trend in the same direction, the khat group did not significantly show a larger Simon effect than the khat-free group (76 vs. 63 ms)—an observation that fits with the previous report of Colzato et al. (2012). In block 2, the interaction tended toward significance, *F*_(1, 27)_ = 3.89, *p* = 0.06, MSE = 373.97, η^2^_*p*_ = 0.13, indicating that khat group showed a smaller Simon effect than the khat-free group (38 vs. 59 ms). This observation is completely in line with the hypothesis tested in the present study.

**Figure 1:**
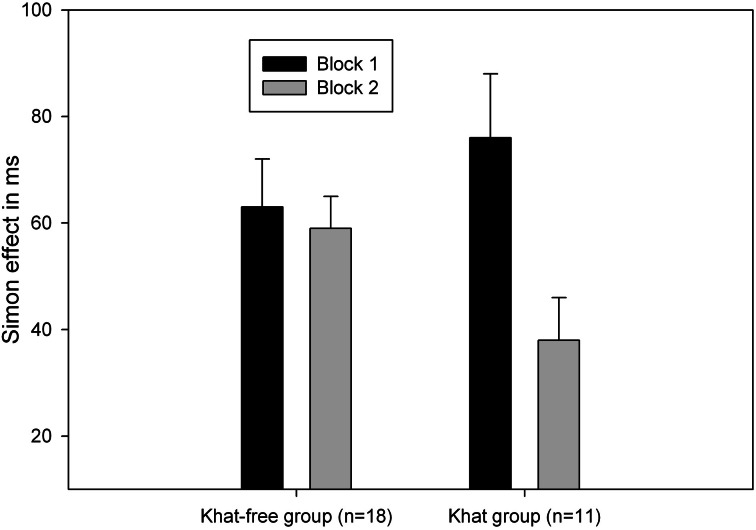
**Mean Simon effect (calculated as the RT difference between non-correspondent and correspondent trials) as a function of block (1 vs. 2) and group (khat users vs. khat-free controls)**. Standard errors of the difference between non-correspondent trials and correspondent trials are represented by the error bars.

Most importantly, ANOVAs performed on the two groups showed a significant Block and Correspondence interaction in the khat group, *F*_(1, 10)_ = 9.49, *p* = 0.01, MSE = 411.54, η^2^_*p*_ = 0.49, but not in the khat-free group, *F* < 1, an indication that, as expected, in khat users the Simon effect was significantly reduced in the second task block (see Figure 1 and Table 3 for the complete data pattern).

**Table 3:** **Performance on the Simon task as a function of block (1 vs. 2), correspondence (correspondent vs. non-correspondent) and group (khat users vs. khat-free controls)**.

**Simon task**	**Block 1**	**Block 2**
**Khat USERS (*n* = 11)**		
Correspondence		
Reaction Times (ms)	550 (32.8)	516 (28.3)
Error Rates (%)	3.0 (0.5)	3.2 (0.7)
Non-correspondence		
Reaction Times (ms)	626 (39.6)	554 (30.6)
Error Rates (%)	8.9 (1.5)	6.6 (1.2)
Simon effect		
Reaction Times (ms)^*^	**76 (11.7)**	**38 (8.2)**
Error Rates (%)	5.9 (1.3)	3.4 (1.0)
**Khat-FREE CONTROLS (*n* = 18)**		
Correspondence		
Reaction Times (ms)	452 (25.6)	417 (22.1)
Error Rates (%)	1.1 (0.4)	1.4 (0.6)
Non-correspondence		
Reaction Times (ms)	514 (30.9)	476 (23.9)
Error Rates (%)	6.3 (1.1)	5.3 (0.9)
Simon effect		
Reaction Times (ms)	**63 (9.2)**	**59 (6.4)**
Error Rates (%)	5.3 (1.0)	3.8 (0.8)

Simon effect is calculated as the Reaction Times and the Error Rates difference between the correspondent and non-correspondent condition. Standard errors of reaction times and error rates are presented in parentheses.

Bold values indicate significant difference between blocks,

* p < 0.05.

### Correlations

To test whether the magnitude of cognitive enhancement in block 2 is proportional to the amount of khat consumed over participants' lifetime, we computed partial correlations between the individual life time khat exposure, last time khat use, hours chewing and number of bundles used in a khat session and the different in Simon effect (in RT) from block 1 to block 2, when controlling for age. No significant correlation was found, *p*'s > 0.44.

## Discussion

This study tested, for the first time, whether acute khat use produces a detectable selective effect on the efficiency to resolve response conflict. As expected, after the intake of khat, users were faster than khat-free controls in selecting the correct response when an alternative response was simultaneously activated. Our results are in line with other studies demonstrating the beneficial cognitive effect of the acute administration of other psychostimulant drugs at moderate dose. Indeed, the acute administration of amphetamine enhances inhibitory mechanisms involved in visual search (Fillmore et al., 2005) and the acute administration of cocaine enhances the inhibition of behavioral responses (Fillmore et al., 2005, 2006). In contrast, the chronic use of the same psychostimulant drugs is associated with detrimental effects on cognition. For instance, long-term users of cocaine found it much more difficult to inhibit responding on stop-trials than non-users (Fillmore and Rush, 2002; Colzato et al., 2007).

We can exclude that the observed pattern of results is produced by the simple activity of chewing because the Simon effect was reduced only in khat users, who performed the task chewing only khat, but not in the control group, who carried out the task while chewing a gum. Moreover, our design allowed us to assess drug-free baseline performance in the first task block took 15 min. to perform, that is, before the onset of action of khat.

We failed to fully match the two groups in term**s** of age: the khat group was significantly older than the khat-free group. However, it is unlikely that this difference accounts for our findings. Indeed, our main result is the opposite of what an age-related effect would lead one to expect: older people show *more*, rather than less, response conflict than younger people (van der Lubbe and Verleger, 2002), while the (older) members of our khat group showed evidence of *lesser* response conflict. In other words, if there was an effect of age it must have worked against our hypothesis.

Another limitation of our study is that it is possible that, in some participants, the onset of action of khat preceded the completion of the first block. Although this cannot be excluded, it would also have worked against our hypothesis, which renders our conclusions rather conservative.

Future research with a greater sample size is needed to extend these preliminary findings. The recent threat of the Dutch government to list khat in the opium act has made research investigating the acute effect of khat very difficult to carry out in the Netherlands. However, the consumption of the drug is still legal in other countries, such as Great Britain, which allows investigating the cognitive effects of acute intake of khat.

Moreover, given the chemical similarity of khat and synthetic cathinones in structure and pharmacological activity, it would be interesting to investigate the cognitive acute effect of mephedrone, a designer drug. It is possible that the acute intake of mephedrone, at least at moderate dose, is associated with similar cognitive facilitation than the acute use of khat. Given that the use of mephedrone has increased in recent years (Wood et al., 2011), it seems of societal relevance to devote more research effort to the functional significance of possible cognitive impact associated with the use of designer drugs.

## Funding

The research of Manuel J. Ruiz is supported by Project EDU 2008-0111: El control inhibitorio de la memoria.

### Conflict of interest statement

The authors declare that the research was conducted in the absence of any commercial or financial relationships that could be construed as a potential conflict of interest.
